# Lack of Recto-Vaginal Septum

**Published:** 1916-03

**Authors:** 


**Lack of Recto-Vaginal Septum.** Frank E. Fox, Fulton, N. Y., Int. Jour. of Surg., Dec. reports a case due to a tear during delivery, the obstetrician mistaking a breech for a face presentation. When seen, the patient was 18 years old, otherwise healthy and well developed. The uterus, which was large, boggy, with cervix raw from continued contact with faeces, was first fixed ventrally. At this time a long chronically inflamed appendix and a cystic right ovary were removed. A plastic operation was successful, even in restoring sphincteric function.

